# Arginase activity in pathogenic and non-pathogenic species of *Leishmania* parasites

**DOI:** 10.1371/journal.pntd.0005774

**Published:** 2017-07-14

**Authors:** Alireza Badirzadeh, Tahereh Taheri, Yasaman Taslimi, Zahra Abdossamadi, Maryam Heidari-Kharaji, Elham Gholami, Baharehsadat Sedaghat, Maryam Niyyati, Sima Rafati

**Affiliations:** 1 Department of Immunotherapy and Leishmania Vaccine Research, Pasteur Institute of Iran, Tehran, Iran; 2 Department of Medical Parasitology and Mycology, School of Medicine, Shahid Beheshti University of Medical Sciences, Tehran, Iran; McGill university, CANADA

## Abstract

Proliferation of *Leishmania* (*L*.) parasites depends on polyamine availability, which can be generated by the L-arginine catabolism and the enzymatic activity of arginase (ARG) of the parasites and of the mammalian hosts. In the present study, we characterized and compared the arginase (*arg*) genes from pathogenic *L*. *major* and *L*. *tropica* and from non-pathogenic *L*. *tarentolae*. We quantified the level of the ARG activity in promastigotes and macrophages infected with pathogenic *L*. *major* and *L*. *tropica* and non-pathogenic *L*. *tarentolae* amastigotes. The ARG's amino acid sequences of the pathogenic and non-pathogenic *Leishmania* demonstrated virtually 98.6% and 88% identities with the reference *L*. *major* Friedlin ARG. Higher ARG activity was observed in all pathogenic promastigotes as compared to non-pathogenic *L*. *tarentolae*. *In vitro* infection of human macrophage cell line (THP1) with pathogenic and non-pathogenic *Leishmania* spp. resulted in increased ARG activities in the infected macrophages. The ARG activities present *in vivo* were assessed in susceptible BALB/c and resistant C57BL/6 mice infected with *L*. *major*, *L*. *tropica* and *L*. *tarentolae*. We demonstrated that during the development of the infection, ARG is induced in both strains of mice infected with pathogenic *Leishmania*. However, in *L*. *major* infected BALB/c mice, the induction of ARG and parasite load increased simultaneously according to the time course of infection, whereas in C57BL/6 mice, the enzyme is upregulated solely during the period of footpad swelling. In *L*. *tropica* infected mice, the footpads' swellings were slow to develop and demonstrated minimal cutaneous pathology and ARG activity. In contrast, ARG activity was undetectable in mice inoculated with the non-pathogenic *L*. *tarentolae*. Our data suggest that infection by *Leishmania* parasites can increase ARG activity of the host and provides essential polyamines for parasite salvage and its replication. Moreover, the ARG of *Leishmania* is vital for parasite proliferation and required for infection in mice. ARG activity can be used as one of the main marker of the disease severity.

## Author summary

Over the past decades, there has been a significant improvement in the understanding of immune responses against infection with pathogenic *Leishmania* spp. and the pathogenesis of cutaneous leishmaniasis (CL) in the mouse models. *Leishmania* parasites infect macrophages that can be activated via two major pathways resulting in classical and alternative activated macrophages which metabolize L-arginine differentially. Classically activated macrophages upregulate the enzyme inducible NO synthase (iNOS) and alternatively activated macrophages upregulate the arginase (ARG). ARG hydrolyzes the conversion of its substrate (L-arginine) to L-ornithine and urea. L-ornithine is a key intermediate substrate for the biosynthesis of polyamines, which are crucial nutrients for cellular processes such as growth, differentiation and proliferation of host cells and *Leishmania* parasites. *Leishmania* parasites may partly activate ARGs and inactivate the NO production by the host cells and enhance parasite survival via depletion of the iNOS substrate (L-arginine) and reduce NO levels. In this study, we tested the activity of this enzyme in the promastigote forms of the pathogenic *L*. *major* and *L*. *tropica*, and non-pathogenic *L*. *tarentolae in vitro* and assessed the differences between the levels of ARG activity in THP1 cells infected with different *Leishmania* spp. Moreover, we investigated the relationship between excessive ARG activity and lesion development in susceptible BALB/c and resistant C57BL/6 mice infected with virulent *L*. *major*, *L*. *tropica* and non-virulent *L*. *tarentolae* parasites and its impact on parasite burden during the development of infection. Our results show that ARG is highly upregulated in all pathogenic promastigotes as compared to non-pathogenic *L*. *tarentolae* and had a negative correlation between production of ARG and NO. We showed that during the development of the infection in susceptible BALB/c and resistant C57BL/6 mice, ARG is induced in both strains of mice infected with pathogenic *Leishmania* but not in non-pathogenic counterparts. These results suggest that ARG activity of *Leishmania* is essential for parasite survival *in vitro* and *in vivo* that directly regulates its growth and replication inside the host cells; therefore, it can be used as a significant marker of disease severity in CL and possible tools for drug designing.

## Introduction

The leishmaniases are neglected parasitic diseases caused by protozoan *Leishmania*, which are present throughout 98 countries [1, 2]. They pose major public health problems mainly in tropical and sub-tropical regions of the globe which affect about 12 million people worldwide [1]. Disease manifestations range from self-healing benign cutaneous to non-healing mucocutaneous leishmaniasis (MCL) and potentially deadly visceral leishmaniasis (VL), in humans [1, 3, 4]. Moreover, post kala-azar dermal leishmaniasis (PKDL) can develop in successfully treated visceral leishmaniasis patients as well as in infected asymptomatic individuals [5]. Flagellated extracellular *Leishmania* promastigotes are inoculated into the skin of their mammalian host by the bite of infected sand flies; mononuclear and polymorphonuclear phagocytes such as neutrophils, dendritic cells (DCs) and more importantly macrophages are recruited to the bite site and phagocytose the promastigotes that differentiate intracellularly into non-motile amastigotes and start multiplying [6–9].

Over the past decades, there has been considerable improvement in the understanding of immune responses against infection with pathogenic *Leishmania* spp. and the pathogenesis of cutaneous leishmaniasis in mouse models. Resistance and susceptibility to *L*. *major* infection are directly connected to the development of two main immune responses (Th1 and Th2) [10]. The Th1 response induced in infected C57BL/6 mice is characterized by localized, non-progressive and healing lesions, whereas a Th2 response mounted by infected BALB/c mice is characterized by large non-healing cutaneous lesions and systemic disease [10, 11]. Host mammalian macrophages are not solely the main host cells; but they are also the main effector cells for *Leishmania* parasites and can be activated via two major pathways resulting in classical and alternative activated macrophages which metabolize L-arginine differently. Classically activated macrophages upregulate the enzyme inducible NO synthase (iNOS) and alternatively activated macrophages upregulate arginase (ARG) [12, 13]. Directly depending on the balance of these two important enzymes, macrophages can be instructed to promote the survival of the intracellular *Leishmania* parasites or to kill them [14]. iNOS catabolizes L-arginine to nitric oxide (NO) and citrulline [12]. NO, a potent inorganic microbicidal agent, is involved in killing of intracellular invading microorganisms such as *Leishmania* [11, 15]. In contrast, ARG induced in alternatively activated macrophages hydrolyzes the conversion of the substrate (L-arginine) to L-ornithine and urea. L-ornithine is a major key intermediate substrate for the biosynthesis of proline, glutamine and polyamines, which are crucial nutrients for cellular processes such as growth, differentiation and proliferation of host cells and *Leishmania* parasites as well [16].

In humans, two isoforms of ARG are named ARG 1 and ARG 2, encoded by two various genes on separate chromosomes, which are genetically different but similar in biochemical characteristics [13, 16]. Interestingly, *Leishmania* parasites, as a lower eukaryote, have their own L-arginine metabolism pathways, and express only one isoform of ARG [17, 18]. Arginase gene sequences from *Leishmania* species such as *L*. *amazonensis* [19], *L*. *mexicana* [18] and *L*. *major* [17] have been characterized. Studies demonstrated that *Leishmania* arginase (*arg*) is a single copy gene that encodes for 330 amino acids. It is essential for parasite growth and surprisingly, expressed in their unique organelle named glycosome [18–21]. ARG expression in the host cells was being utilized for generation of polyamines. Indeed, the intracellular growth of *Leishmania* parasites in infected cells can be controlled by inhibiting ARG by means of Nω-hydroxy-arginine (OH-arg) or Nω-hydroxy-nor-L-arginine (nor-NOHA) as physiological inhibitors of ARG [22].

In spite of ARGs expression by both the mammalian macrophages and the *Leishmania* parasites, it is not clear whether one or both ARGs are essential for parasite growth in the host cells [11]. Studies showed that parasite-derived ARG may indirectly trigger the production of ARGs and reduces arginine pools of the host cells and then enhance parasite survival via local depletion of the iNOS substrate L-arginine and reduced NO levels [11, 23]. Therefore, in the present study, we assessed and compared the ARG activity in pathogenic *L*. *major* (MRHO/IR/75/ER), *L*. *tropica* (MOHM/IR/09/Khamesipour-Mashhad) and non-pathogenic *L*. *tarentolae* Tar II (ATCC 30267) at genomic and enzymatic activity levels in promastigote forms of the parasites *in vitro*. Moreover, we tested the differences between the levels of ARG activity in macrophages (THP1) infected with different *Leishmania* spp. Our results demonstrated a negative correlation between production of ARG and NO. Others have been recently demonstrated that uncontrolled replication of *L*. *major in vivo* at the infection site in susceptible BALB/c mice correlates with abnormally high activity of ARG [10, 24]. Therefore, here, we investigated the relationship between excessive ARG activity and lesion development in susceptible BALB/c and resistant C57BL/6 mice infected with virulent *L*. *major*, *L*. *tropica* and non-virulent *L*. *tarentolae* parasites and its impact on parasite burden during the course of infection.

## Methods

### Ethics statement

The current study was approved by the Human and Animal Research Ethics Committee of Pasteur Institute of Iran (ID 8916, May 2013), based on the Specific National Ethical Guidelines for Biomedical Research issued by the Research and Technology Deputy of Ministry of Health and Medicinal Education (MOHME) of Iran (issued in 2005). In this study, all efforts were made to minimize animal suffering within the course of our study.

### Mice

6–8 week old female BALB/c and C57BL/6 mice weighting 20±5g were purchased from the breeding stock maintained at the Pasteur Institute of Iran (Tehran, Iran) and maintained in ventilated cages for this study. Throughout the experiment, all mice (BALB/c and C57BL/6) were kept in an air conditioned controlled animal care facility (23±2°C; humidity: 50–60%) and 12 hours light-dark cycles, with free access to standard rodent food and appropriate tap water.

### Parasites and inoculation of mice with pathogenic and non-pathogenic *Leishmania*

Pathogenic species of *L*. *tropica* (MOHM/IR/09/Khamesipour-Mashhad), *L*. *major* (MRHO/IR/75/ER) and non-pathogenic *L*. *tarentolae* Tar II (ATCC 30267) were used in the current study. The *L*. *tropica* (MOHM/IR/09/Khamesipour-Mashhad) and the *L*. *major* (MRHO/IR/75/ER) were provided as gifts from Dr. A. Khamesipour (Center for Research and Training in Skin Diseases and Leprosy, Tehran University of Medical Sciences, Tehran, Iran) and Dr. E. Javadian (School of Public Health, Tehran University of Medical Sciences, Iran) respectively. The *L*. *tarentolae* Tar II (ATCC 30267), was generously provided by Dr. B. Papadopoulou (Research Centre in Infectious Disease, CHUL Research Centre and Department of Microbiology, Infectious Disease and Immunology, Laval University, Quebec, Canada). The pathogenic parasites were maintained in a virulent state by continuous passage in BALB/c mice. The isolated homogenized lymph nodes (LNs) from BALB/c mice were cultured at 26°C in Schneider’s Drosophila medium (Sigma, Darmstadt, Germany) pH 7.4, supplemented with 10% heat-inactivated fetal calf serum (HI-FCS) (FCS, Gibco, UK), 40 mM HEPES, 2 mM, 0.1 mM adenosine, 2 mM L-glutamine, 0.5 μg/ml hemin (all from Sigma, Germany) and 50 μg/ml gentamicin (Biosera, France). Non-pathogenic *L*. *tarentolae* promastigotes were grown at 26°C in identically supplemented Schneider’s Drosophila medium and were allowed to multiply until they reached a density of 2 × 10^8^ parasites/ml. Mice were infected subcutaneously in their hind footpad with 50 μl of 2 × 10^6^, 2 × 10^7^ and 2 × 10^7^ stationary phase promastigotes of *L*. *major*, *L*. *tropica* and *L*. *tarentolae* respectively. The development of the lesion was monitored and recorded for each mouse weekly by measuring the diameter of the footpad swelling using metric caliper. For analyzing the course of infection caused by each parasite species, 4 groups with a total number of 18 mice were used and sacrificed (n = 3 mice per group) at 1, 3, 5, 7 and 10 weeks post-infection determine parasite load and ARG activity at the site of infection and in the draining lymph nodes.

### THP1 cells culture

The immortalized human monocyte cell line THP1 (ATCC TIB-202 TM) was cultured in RPMI-1640 supplemented with 10% FCS at 37°C in a 5% CO2 incubator. To induce adherent and differentiated cells, THP1 cells were counted and then treated with 5 μg/ml phorbol 12-myristate 13-acetate (PMA) (Sigma, Germany) [25]. After 24 hours of incubation, PMA-treated THP1 cells were differentiated, and for macrophage infection, stationary (5 days) phase promastigotes of all species were used. Total of 5×10^5^ PMA-treated THP1 cells were infected with stationary phase promastigotes of *L*. *major*, *L*. *tropica* and *L*. *tarentolae* at a multiplicity of infection (MOI) of 1:10, and then was cultured for 48 h at above conditions. All free parasites were removed by washing with serum-free RPMI-1640 medium. The supernatants and cell lysates were harvested for the assessment of NO and ARG activity, respectively [26].

### Cloning and sequencing of the arginase gene of *Leishmania* spp.

Genomic DNA was purified from log phase promastigotes of the *Leishmania* spp. (2×10^8^ parasites/ml) by using the GF-1 Nucleic Acid extraction kit (GF-1, Vivantis, Canada) according to the manufacturer's guidelines. The DNA concentration and purity was quantified using a NanoDrop (ND-1000) spectrophotometer. The *arg* sequence from *L*. *major* Friedlin strain (GenBankTM accession number NC_007284) was applied to design primers to amplify the *arg* from the genomic DNA of *L*. *major*, *L*. *tropica* and *L*. *tarentolae* by the polymerase chain reaction (PCR). The sense and anti-sense primer sequences were as follows: 5′-CT CGA G**AT G**GA GCA CGT GCA GCA GTA C-3′, 5′-CGC TAG C**CT A**CA GCT TGG CGT CCT TAC G-3′, respectively. The primers encompassed the initiation methionine codon and a stop codon (bold letter) before by the *Xho*I and *Nhe*I restriction site, respectively as underlined. The PCR product sizes were ~ 1000 bp. PCR reactions were carried out in the Taq buffer, using 1 U Taq DNA Polymerase (Roche, Germany) supplemented with MgCl_2_ (1.5 mM), dNTPs (200 μM) and primers (400 nM each) in a total volume of 50 μl. For amplification, DNA was denatured at 95°C for 5 min, followed by 35 cycles of 95°C for 1 min, 60°C for 30 s and 72°C for 50 s; and a final extension cycle of 72°C for 20 min. Purification of the PCR products were done by using the Wizard SV Gel and PCR Clean-Up System (Promega, USA) and cloned into pCR2.1 vector (Invitrogen, Groningen, the Netherlands). DNA sequencing was applied using T7 and SP6 primers by the dideoxy nucleotide chain termination method of Sanger et al. [27].

### Alignment and phylogenetic analysis of nucleotides and amino acids sequences

All nucleotide sequences are deposited in the GenBank database under the accession numbers KU641750 (*L*. *major* MRHO/IR/75/ER), KU641752 (*L*. *tarentolae* Tar II ATCC 30267) and KU641753 (*L*. *tropica* MOHM/IR/09/Khamesipour-Mashhad). The analysis were carried out using the BLAST analysis for amino acid and nucleic acid pairwise sequence alignment [28] and ClustalW programs for multiple sequence alignments [29] as implemented in BioEdit software, version 7.2.5 [30] and improved in MAFFT [31]. Finally all sequences were aligned and a phylogenetic tree was constructed by MEGA v5.05 software [32].

### Gene transcription analysis by quantitative real-time PCR (qPCR)

Total RNA was extracted from logarithmic and stationary growth phases of *L*. *tropica* (MOHM/IR/09/Khamesipour-Mashhad), *L*. *major* (MRHO/IR/75/ER), and *L*. *tarentolae* Tar II (ATCC 30267) (2×10^8^ parasites/ml) using RNeasy Plus Mini Kit (Qiagen, Valencia, CA) following the manufacturer’s recommendations. Reverse transcription was done with a random primer protocol (Fermentas, M-MuLV RT) and Omniscript RT Kit (Qiagen, Valencia, CA). The obtained cDNA was diluted in H_2_O and utilized in qPCR. Results of the PCR analyses were normalized against the *Leishmania* housekeeping gene 18S rRNA. The primers designed in the current study were as follows: RT-Arg (Forward: 5′-TCC CGA GTG CTT TTC GTG G-3′, Reverse: 5′-TCC ACG TGA TGC ATG CTG AA -3′), 18S rRNA (Forward: 5′-GGG AAA CCC CGG AAT CAC AT-3′, Reverse: 5′- GGT GAA CTT TCG GGC GGA TA-3′). Real-time quantitative PCR (qPCR) analyses were performed with Applied Biosystem 7500 real time PCR system (Applied Biosystems, FosterCity, CA, USA). The comparative method was used to analyze gene expression. ARG threshold (C_t_) values were normalized to the *Leishmania* housekeeping gene 18S rRNA expression as determined by ∆C_t_ = C_t (target gene) ¯_ C_t (18S rRNA control)_. Fold change was quantified by using 2^-∆∆Ct^, where ∆∆C_t_ = ∆C_t (target) ¯_ C_t (18S rRNA control)_ [33].

### Quantification of parasite load in lymph node (LN)

Three mice from each group were sacrificed at 1, 3, 5, 7 and 10 weeks post-infection and genomic DNA was isolated from draining LN using DNeasy Blood & Tissue kit (Qiagen). The parasite burden (*L*. *major*, *L*. *tropica* and *L*. *tarentolae*) in the infected LN was determined by Real time PCR [34]. The sequences of the primers targeting a region of kinetoplastid minicircle DNA were as follows: RV1 and RV2 primers for *L*. *major* and *L*. *tarentolae* forward: 5′-CTTTTCTGGTCCCGCGGGTAGG-3′, reverse: 5′-CCACCTGGCCTATTTTACACCA-3′; kDNA1 primer for *L*. *tropica* forward: 5′-GGGTAGGGGCGTTCTGC-3′, 5′-TACACCAACCCCCAGTTTGC-3′ [35]. The absolute copy number of the target sequence was measured by using Applied Biosystem 7500 real time PCR system (Applied Biosystems, FosterCity, CA, USA). *L*. *major*, *L*. *tropica* and *L*. *tarentolae* genomic DNA was used in 10-fold dilutions corresponding to 2×10^8^ parasites and used in real time PCR to draw the standard curve.

### Measurement of arginase activity

The enzymatic activity of ARG was measured in the promastigotes, in lysates of differentiated THP1 cells infected by stationary phase promastigotes (MOI of 1:10) and in tissue homogenate from infected mice. The arginase activity was determined by measuring the conversion of L-arginine to L-ornithine and urea using the micro-method described elsewhere [12, 21, 36, 37]. Briefly, 25 μl of cell lysates was solubilized with 25 μl of lysis buffer containing: 0.1% Triton x-100, 10 mM MnCl2 and 50 mM Tris-HCl (pH 7.5). Arginase was activated by heating for 7 min at 56°C. L-arginine hydrolysis was done by incubating the activated lysates with 50 μl of L-arginine (pH 9.7) at 37°C for 60 min. The reaction was stopped by the addition of 400 μl acid solution (H_2_SO_4_ (96%)/H_3_PO_4_ (85%)/H_2_O (1:3:7, v/v/v). Urea concentration was measured at 540 nm after addition of 20 μl of α-isonitrosopropiophenone (ISPF, dissolved in 100% ethanol, Sigma) using a spectrophotometer (TECAN, USA) followed by heating at 100°C for 45 min. One unit of enzyme (ARG) activity is defined as the amount of enzyme that catalyzed the formation of one μmol of urea per 60 second.

### Protein determination

The total protein concentrations of the cell lysates were quantified using a bicinchoninic acid (BCA) protein assay kit (Thermo Scientific Pierce Chemical Co, Massachusetts, USA) and serially diluted bovine serum albumin (BSA) as standards [38]. Following 30 min incubation at 37°C, the optical density (OD) was determined at 540 nm.

### Measurement of nitrite production

Nitrite, as a stable end product of the catabolisms of L-arginine by iNOS was quantified in the supernatants of differentiated THP1 cells 48h after infection with *Leishmania* spp (MOI of 1:10) as an indicator of nitric oxide production. Total of 100 μl of culture supernatant was collected from each well and subsequently mixed with an equal volume of Griess reagent kit (Promega) [0.1 N (1-napthyl) ethylenediamine dihydrochloride, 1% sulfanil amide in 5% H_3_PO_4_] [39–42]. Absorbance of the colored complex was measured at 550 nm. The NO concentration of each sample was extrapolated based on the standard curve plotted with serial dilutions of NaNO_2_ in culture medium, covering a concentration range from 1–300 μM.

### Statistical analysis

Statistical analysis was done using Graph-Pad Prism 6.0 for Windows (Graph-Pad Prism, San Diego, California, USA). For *in vitro* and *in vivo* evaluation, t-test or one-way ANOVA was applied to analyze ARG assay, NO production and footpad swelling in the current study. The p-values less than 0.05 (P value <0.05) were considered statistically significant. Data were presented as mean ± standard deviation (SD).

## Results

### Analysis of ARG sequences

DNA sequence analysis of the two pathogenic *Leishmania* parasite species and the non-pathogenic *L*. *tarentolae* revealed an open reading frame (ORF) consisting of 990 and 987 nucleotides respectively, which encoded 330 amino acids (S1 Fig). The sequenced genes were translated to amino acids and compared with GenBank sequences of three reference species of *Leishmania* including: *L*. *major* Friedlin (XM_003722493.1), *L*. *infantum* JPCM5 (XM_001468931.1) and *L*. *mexicana* (MNYC/BZ/62/M379) (Table 1). The multisequence alignment as shown in S1 Fig elucidated that the *L*. *major*, *L*. *tropica* and *L*. *tarentolae* ARG ORFs are 99%, 98% and 88% identical to the predicted ARG proteins of *L*. *major* Friedlin (XM_003722493.1), 98%, 97% and 89% to *L*. *infantum* JPCM5 (XM_001468931.1) and 96%, 95% and 88% to *L*. *mexicana* ARG, respectively. The *L*. *major* and *L*. *tropica* ARG proteins are 43% and 38% identical to the two isoforms of human (*Homo sapiens*) ARG enzymes, ARG 1 and ARG 2, respectively. *L*. *tarentolae* ARG is 41% and 38% identical to the ARG 1 and ARG 2, respectively. Furthermore, the *L*. *major* and *L*. *tropica* ARG proteins are 42% and 39% identical to the two isoforms of mice (*Mus Musculus*) ARG enzymes, ARG 1 and ARG 2, respectively. *L*. *tarentolae* ARG is 40% and 39% identical to the ARG 1 and ARG 2, respectively (S1 Fig). Although, the total amino acid sequences homology between mammalian (human or mice) and *Leishmania* ARGs is solely 40% in average, residues essential for L-arginine as a substrate and inhibitors binding are highly conserved between these two ARGs. One of the major differences between mammalian and *Leishmania* ARGs is the presence of two non-conserved amino acids that creates distinct channel like domains by the existence of methionine (239) in *Leishmania* instead of histidine (228) in human. It was suggested that these differences between the two ARGs in the vicinity of the active sites or catalytic centers are not conserved and can be exploited in parasites ARG inhibitors. Active sites or catalytic centers of both are vital for protein function and processing [19, 43].

**Table 1:** Blast alignment of sequenced *Leishmania* spp. ARG and three retrieved reference *Leishmania* ARG sequences of the GenBank in DNA and amino acids level.

Sequenced Species	DNA (Reference sequences) (%)			Amino acid (Reference sequences) (%)		
	*L*. *major* Friedlin	*L*. *infantum* JPCM5	*L*. *mexicana MNYC*	*L*. *major* Friedlin	*L*. *infantum* JPCM5	*L*. *mexicana MNYC*
*L*. *major* (MRHO/IR/75ER)	99	97	95	99	98	96
*L*. *tropica* (MOHM/IR/09/Khamesipour-Mashhad)	97	97	96	98	97	95
*L*. *tarentolae* Tar II (ATCC 30267)	89	89	89	88	89	88

The translated proteins were nearly identical to its reference sequences of *L*. *major* Friedlin, *L*. *infantum* JPCM5 and *L*. *mexicana* equivalents, differing only one amino acid in sequenced *L*. *major* and *L*. *tropica* as well as two amino acids in *L*. *tarentolae* proximal to the COOH terminus. A unique and notable feature of *Leishmania* spp. ARG protein is the existence of a glycosomal (peroxisomal) targeting signal SKL and AKL, a tripeptide located in the deduced COOH terminus [18, 44]. Like other *arg* of *Leishmania* species [17, 18, 21], the *L*. *major*, *L*. *tropica* and *L*. *tarentolae arg* genes encode this COOH terminus tripeptide that mediate the translocation of the protein to the glycosome. Importantly, the ARG of *L*. *major* and *L*. *tropica*, two Old World *Leishmania* species, and also *L*. *tarentolae*, encode an AKL (alanine-lysine-leucine) COOH terminus tri-peptide, while the ARG from New World *Leishmania* spp. like *L*. *amazonensis* and *L*. *mexicana*, encompass an SKL (serine-lysine-leucin) COOH terminus tripeptide. Both tri-peptides (AKL and SKL) are the most typical topogenic signature for targeting protein to the glycosome in *Leishmania* spp. (S1 Fig) [45–47].

### Phylogeny of *Leishmania* arginase

Gene-by-gene alignment, comparison and analysis was done for all sequenced *arg* of *Leishmania* parasites to prepare data sets for phylogenetic analyses. To determine the relationship of the lately sequenced *arg* genes with known sequences, an evolutionary tree was constructed by using the maximum-likelihood (ML) method [32]. The phylogenetic relationship among 10 *Leishmania* parasites, two *Homo sapiens* and two *Mus musculus arg* genes is presented in S2 Fig. Among the three sequenced *Leishmania arg* genes, *L*. *major* and *L*. *tropica* were found to be more similar to the reference *L*. *major* Friedlin strain and have similar length in the phylogenetic tree and all nodes supported by high bootstrap values. Our data show that *L*. *tarentolae* is less similar to other *Leishmania* parasites. ARG of distant organisms such as *H*. *sapiens* and *M*. *musculus* was partly different from *Leishmania* parasites ARG (30–40%), and according to S2 Fig the phylogenetic relationships among human and mice ARG 1 and ARG 2 and *Leishmania* parasites were uncertain because their nodes presented low bootstrap values.

### Arginase activity in *Leishmania* promastigotes

**Fig 1:**
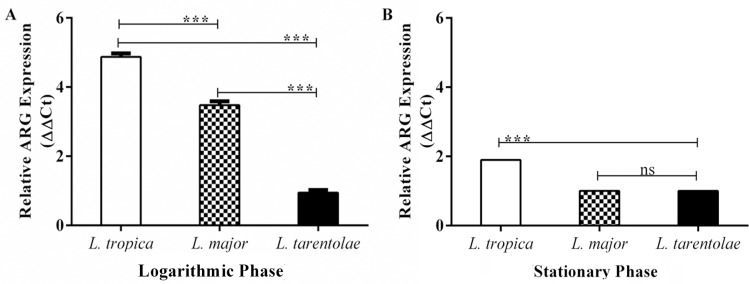
Real-time qPCR analysis of total RNA from cultured species of *Leishmania* promastigotes. Arginase transcript levels were quantified in extracted total RNAs of (A) logarithmic and (B) stationary growth phase of promastigotes (2×10^8^ parasites/ml) for each parasite separately. Data reported are those of duplicate samples, and the experimental procedure was repeated at least three times with identical outcomes. Error bars are SD (**p ≤ 0.01, ****p ≤ 0.001 and *****p ≤ 0.0001).

**Fig 2:**
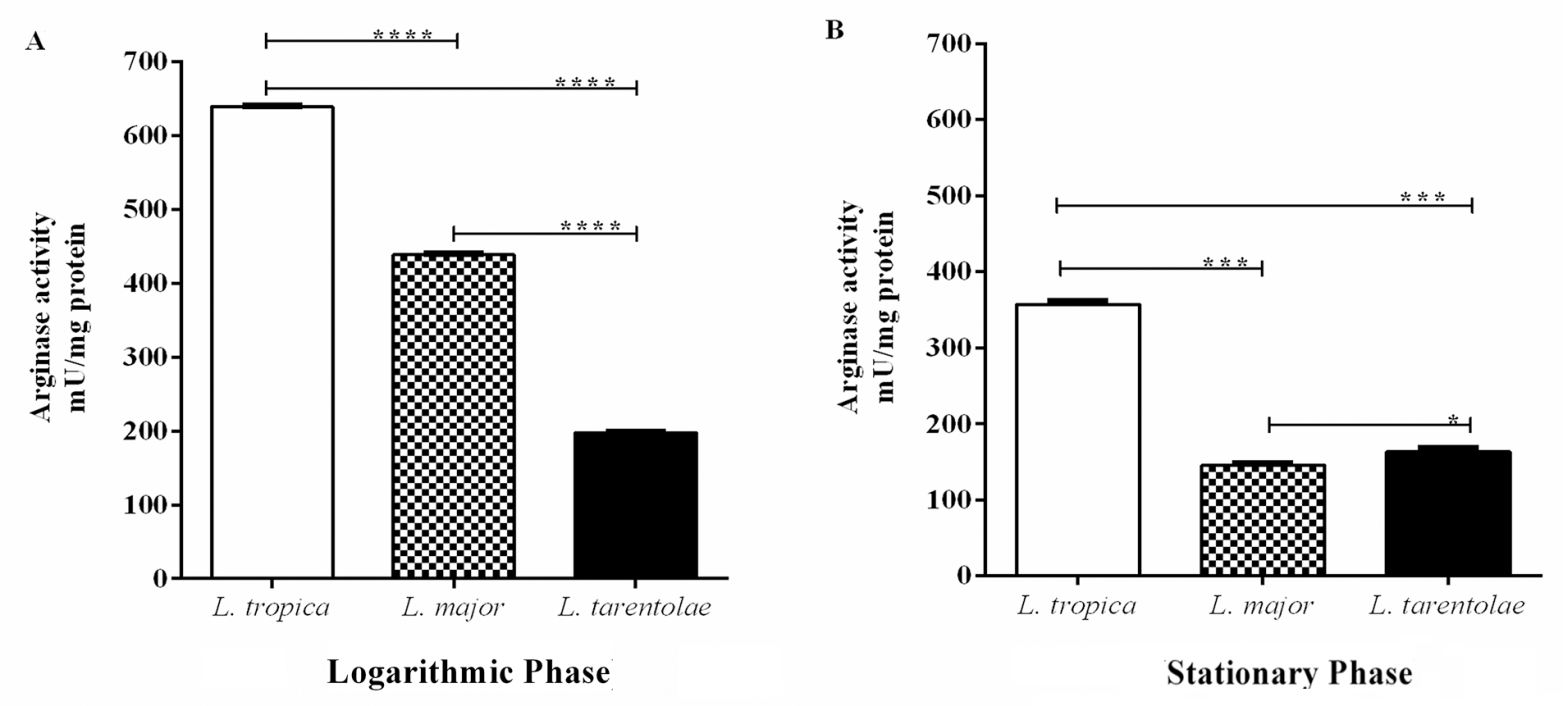
Arginase activities in the cultured species of pathogenic and non-pathogenic *Leishmania* promastigotes. Promastigotes from (A) logarithmic and (B) stationary growth phases were obtained from cultures (2×10^8^ parasites/ml). Data reported are those of duplicate samples, and the experimental procedure was repeated at least three times with identical outcomes. Error bars are SD (**p ≤ 0.01, ****p ≤ 0.001 and *****p ≤ 0.0001).

It has been previously shown that *Leishmania* spp. expresses their own ARG enzyme [12, 23, 48]. It is vital for their growth and differentiation since knocking out the *arg* gene on both alleles of the parasite was fatal and became auxotrophic for polyamines [18, 19, 43, 47]. High-quality total RNAs were extracted from all the species of *Leishmania* parasites we investigated in this study. It is crucial to analyze the exact transcript levels of ARG in promastigotes; therefore, we quantified the ARG transcript levels of promastigotes at the logarithmic (Fig 1A) and stationary (Fig 1B) growth phases of both pathogenic and non-pathogenic *Leishmania*. As shown in Fig 1, *L*. *tropica* expressed the highest transcript levels of ARG in the both logarithmic and stationary growth phases as compared with *L*. *major* and *L*. *tarentolae* (p<0.05). Although significant ARG transcript levels were detected in the pathogenic *Leishmania* species, it is low in the non-pathogenic *L*. *tarentolae* (p<0.05). We also measured the enzymatic activity of ARG in both pathogenic and non-pathogenic *Leishmania* promastigotes in the logarithmic and stationary growth phases. Our experiments revealed that significant ARG activity was detected in the both pathogenic and non-pathogenic *Leishmania* promastigotes (Fig 2). As shown in this figure, ARG activities in the logarithmic phase (Fig 2A) of the parasites are two-fold higher than in the stationary phase (Fig 2B), and there was significantly higher ARG activity in *L*. *tropica* and *L*. *major* as compared to non-pathogenic *L*. *tarentolae* (p<0.05). In the logarithmic phase, the highest and lowest ARG activities were expressed by *L*. *tropica* (638.63 mU/mg protein) and *L*. *tarentolae* (197.45 mU/mg protein) respectively. In contrast, in the stationary phase *L*. *tropica* promastigotes exhibited the highest specific activity of ARG (420.47 mU/mg protein) and *L*. *major* the lowest (145.34 mU/mg protein) (p<0.05).

### Arginase activity and NO production in the supernatant of *Leishmania* infected macrophage cell line (THP1)

**Fig 3:**
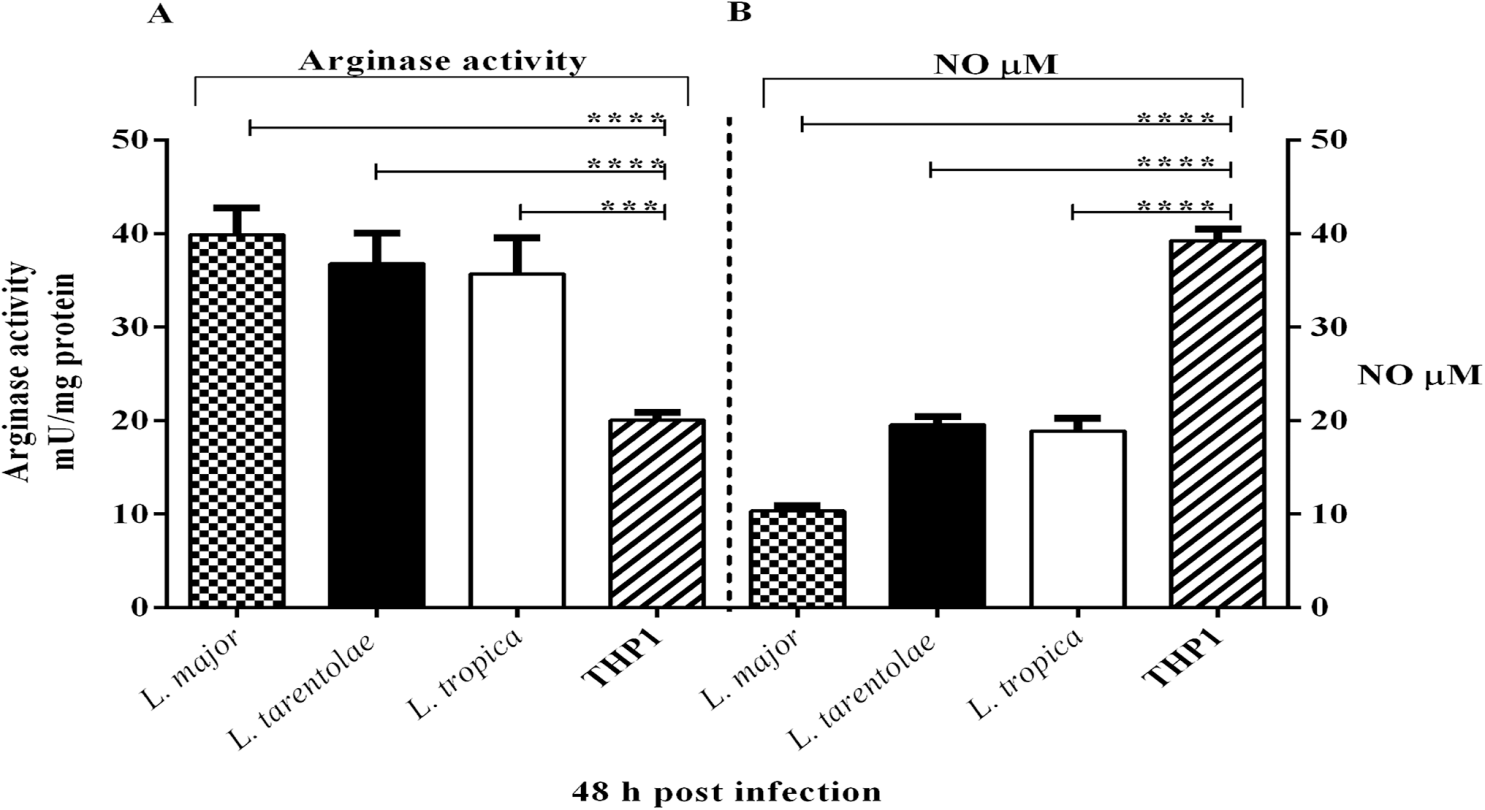
Comparative levels of ARG activity and NO production in infected THP1 cells. Cells from the human macrophages cell line (THP1) were infected with stationary growth phase of pathogenic and non-pathogenic *Leishmania* promastigotes (2×10^8^ parasites/ml) with a MOI of 1:10 and after 48 hours post-infection the amount of (A) ARG and (B) NO released into culture supernatants was measured. Data reported are those of duplicate samples, and the experimental procedure was repeated at least three times with identical outcomes. Error bars are SD (**p≤ 0.01, ****p≤ 0.001 and *****p≤ 0.0001).

ARG and iNOS are two inducible enzymes that crucially influence the *Leishmania* infection outcomes: the induction of ARG results in the catabolism of L-arginine into urea and ornithine; the latter is further catabolized into polyamines that are required for parasite growth while iNOS oxidizes L-arginine in a two steps process into NO, a metabolite responsible for *Leishmania* parasite clearance. Both enzymes are induced in macrophages and share L-arginine as a substrate and are crucial indicators for the disease outcome [14, 49]. As shown in Fig 3A, ARG activities in infected THP1 cells increased approximately two-fold as compared to uninfected THP1 cells (p<0.05). No significant differences were observed among the pathogenic and non-pathogenic species in the activity of ARG (p<0.05). As illustrated in Figs 2 and 3A, we could detect high activity of ARG in the promastigotes (10-fold higher) compared to *Leishmania* infected THP1. Our findings are in agreement with other studies elucidating that ARG is vital for growth and proliferation of *Leishmania* promastigotes but not intracellular amastigotes [47]. Studies have shown that NO production was detected in the supernatants of *Leishmania* infected macrophages [50–52], and we obtained similar results in infected THP1 cells. As shown in Fig 3B, THP1 cells infected by pathogenic and non-pathogenic *Leishmania* produced lower levels of NO as compared to non-infected THP1 cells (p<0.05). As expected, our data show a direct relationship between ARG activity and NO levels, when ARG activity was increased in infected macrophages, the NO production was decreased (Fig 3).

### ARG activity and parasite load in *L*. *major* infected mice

**Fig 4:**
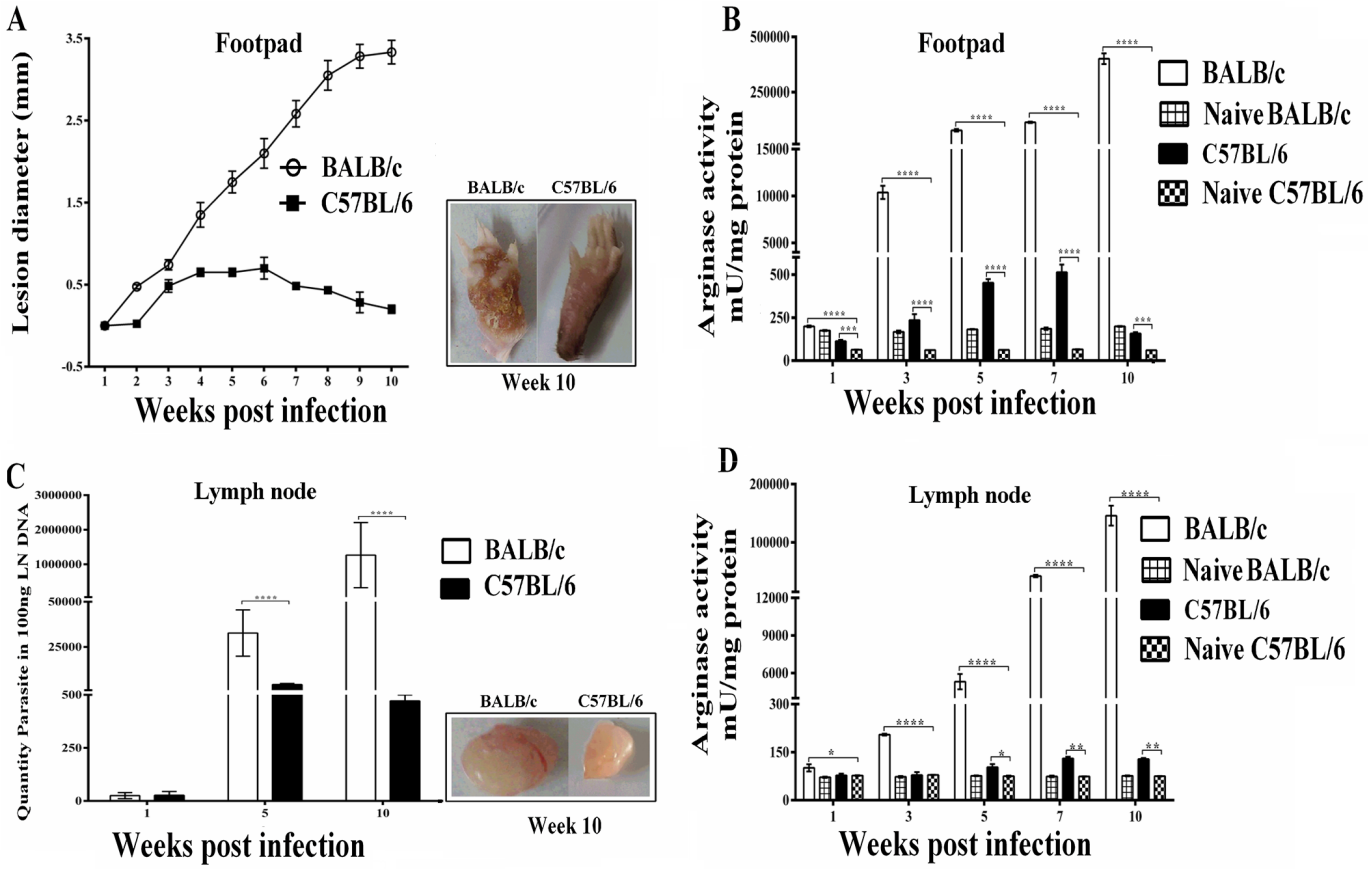
Lesion development, ARG activity and parasite burden in *L*. *major* infected mice in footpads and draining lymph nodes. Groups of susceptible BALB/c and resistant C57BL/6 mice were infected with 2×10^6^ metacyclic *L*. *major* promastigotes in the left hind footpad. (A) The lesion size was monitored by measuring the increase in footpad thickness and width weekly by using a caliper. At various times after infection (ending at 10 week) mice were sacrificed and ARG activity was determined in (B) footpads and (D) lymph nodes. (C) Parasite number was quantified in draining lymph nodes at 1, 5 and 10 weeks after infection. Data reported are those of duplicate samples, and the experimental procedure was repeated at least two times with similar outcomes. Error bars are SD (**p≤ 0.01, ****p≤ 0.001 and *****p≤ 0.0001).

To determine the effects of pathogenic *L*. *major* on the virulence and development of CL in murine models, genetically susceptible BALB/c and resistant C57BL/6 mice were infected into the left hind footpad and monitored weekly for the onset and development of lesion over time. Fig 4A shows lesion development in the footpad of *L*. *major* infected BALB/c and C57BL/6 mice. Visible footpad swelling was observed starting at 2 weeks post infection and increased more rapidly in BALB/c mice, which developed progressive non-healing lesions and disfigured lesions. In sharp contrast, in resistant C57BL/6 mice, footpad swelling increased less pronounced until week 5 but decreased progressively after 6 weeks post-infection (Fig 4A).

It has been previously shown that increased lesion size of *L*. *major* infected BALB/c mice correlated with higher ARG activity [12]. Therefore, we hypothesized that ARG activity at the sites of pathology would be different in *L*. *major* infected susceptible and resistant strains of mice. In both strains of *L*. *major* infected mice the ARG activities were significantly higher than those quantified in naive un-infected mice (Fig 4B). The ARG activity quantified in footpad homogenates showed that the level of ARG activity were significantly higher during the course of lesion development in susceptible BALB/c mice, whereas lower ARG activity were present in the footpad homogenates of infected C57BL/6 mice (p<0.05) (Fig 4B). It is noteworthy that the uncontrolled parasite growth in *L*. *major* infected BALB/c mice correlated with increasing raise in ARG activity. In resistant C57BL/6 mice, the ARG activity declined and almost reached background levels at the time of healing and resolution of lesions when the parasite growth was controlled.

To answer whether parasite growth and enhanced ARG activities were restricted to the local site of parasite inoculation we quantified parasite burden as well as the ARG activity levels during the course of infection in BALB/c and C57BL/6 mice in the nearest draining LN. As shown in Fig 4C and 4D parasite load and also ARG activity in the draining LN was also significantly higher in susceptible BALB/c mice than in resistant C57BL/6 mice.

### ARG activity and parasite load in *L*. *tropica* infected mice

**Fig 5:**
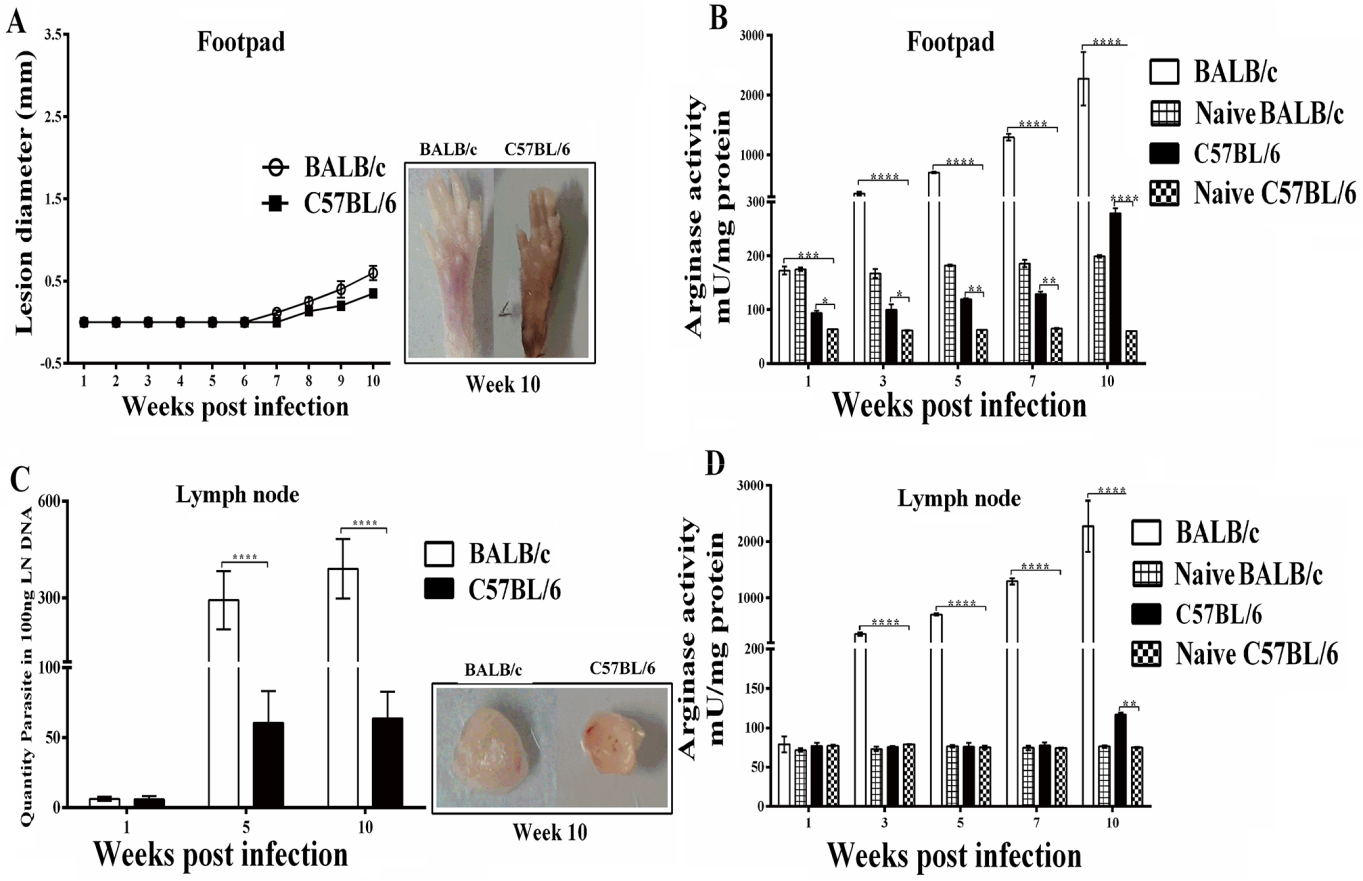
Lesion development, ARG activity and parasite burden in *L*. *tropica* infected mice in footpads and draining lymph nodes. Groups of susceptible BALB/c and resistant C57BL/6 mice were infected with 2×10^7^ metacyclic *L*. *tropica* promastigotes in the left hind footpad. (A) The lesion size was monitored by measuring the increase in footpad thickness and width weekly using a caliper. At various times after infection (ending at 10 week), mice were sacrificed and ARG activity was determined in (B) footpads and (D) lymph nodes. (C) Parasite number was quantified in draining lymph nodes at 1, 5 and 10 weeks after infection. Data reported are those of duplicate samples, and the experimental procedure was repeated at least two times with similar outcomes. Error bars are SD (**p≤ 0.01, ****p≤ 0.001 and *****p≤ 0.0001).”

To investigate the course and progression infection with pathogenic *L*. *tropica* BALB/c and C57BL/6 mice were infected into the left hind footpad and the development of lesions was monitored at regular intervals post infection (Fig 5A). The patterns of footpad swelling in *L*. *tropica* infected BALB/c and C57BL/6 mice developed with almost similar kinetics and only minor swelling at 6 and 7 weeks post-infection were observed (Fig 5A).

Earlier studies showed that lesion development was slow in *L*. *tropica* infected C57BL/6 mice but was persistent with chronic pathology at the site of infection [53]. Therefore, to determine whether the effect of chronic *L*. *tropica* infection in mice may be associated with higher ARG activity at the site of pathology, we quantified ARG activity in footpad homogenates. The results showed significantly higher ARG activity in the lesions of infected BALB/c mice than in C57BL/6 mice infection (p<0.05) (Fig 5B); although, both strains of *L*.*tropica* infected mice were able to contain lesion growth. Compared to *L*. *major* infected mice, footpad swelling and ARG activity in the *L*. *tropica* infected mice was significantly lower (Fig 5A and 5B).

To determine whether parasite numbers regulate ARG activities of the infected host, we quantified parasite burden and the ARG activities during the course of chronic *L*. *tropica* infection in the draining LNs of BALB/c and C57BL/6 mice at distinct time post infection. As shown in Fig 5C and 5D both parasite loads as well as ARG activity in the draining LN were significantly higher in BALB/c mice than in C57BL/6 mice. In BALB/c, which are very susceptible to *L*. *major* infection, *L*. *tropica* infection progressed with same pattern as in C57BL/6 mice, with a peak parasite load attain after 10 weeks (almost 400 parasites per LN). Importantly, the *L*. *tropica* did not grow uncontrolled in the LNs of BALB/c; the growth of *L*. *tropica* in LN of C57BL/6 mice, progressed very slowly and attained a peak number of almost 70 per LN (Fig 5C).

### Non-pathogenic *L*. *tarentolae* infection in mice does not lead to increased ARG activity

**Fig 6:**
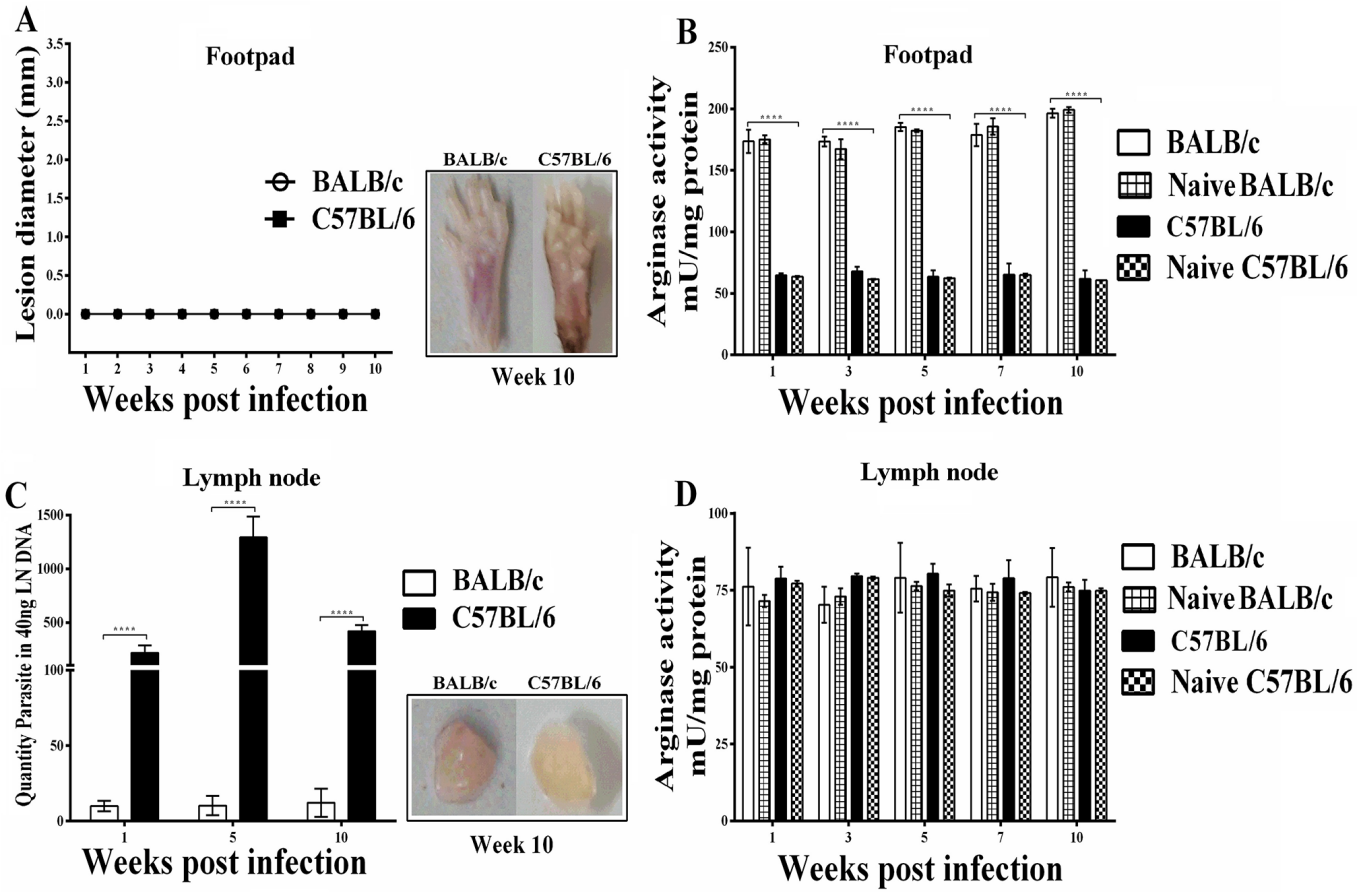
Lesion development, ARG activity and parasite burden in mice injected with non-pathogenic *L*. *tarentolae*. Groups of susceptible BALB/c and resistant C57BL/6 mice were infected with 2×10^7^ metacyclic *L*. *tarentolae* promastigotes in the left hind footpad. (A) The lesion size was monitored by measuring the footpad thickness and width weekly using a caliper. At various times after infection (ending at 10 week) mice were sacrificed and ARG activity was determined in (B) footpads and (D) lymph nodes. (C) Parasite number was quantified in draining lymph nodes at 1, 5 and 10 weeks after infection. Data reported are those of duplicate samples, and the experimental procedure was repeated at least two times with similar outcomes. Error bars are SD (**p≤ 0.01, ****p≤ 0.001 and *****p≤ 0.0001).

It has been recently shown in experimental models as well as in CL patients that high activity of ARG, a hallmark of non-healing persistent leishmaniasis, is mainly increased at the site of pathology [24, 54]. Given that, we tested the hypothesis that non-pathogenic *L*. *tarentolae*, although they can infect mouse macrophages [55], could not induce disease due to the inability to upregulate ARG activity at the site of infection and in the draining LN. Therefore, susceptible BALB/c and resistant C57BL/6 mice were infected into the left hind footpad and monitored for the development of lesion at regular intervals post infection. As shown in Fig 6A and 6B, BALB/c and C57BL/6 mice developed neither lesions nor ARG activity at the site of infection and no significant differences were seen between naive control groups and infected test groups in both strains of mice.

We next quantified parasite load in the draining lymph nodes (LN) at the site of pathology at different times post infection to better understand the infection process. We unexpectedly found that, in C57BL/6 mice parasite load was higher than BALB/c whereas there was no significant difference in ARG activities in infected mice (Fig 6C and 6D).

Similarly, we checked the ARG activity due to non-pathogenic *L*. *tarentolae* in draining LNs of BALB/c and C57BL/6 mice. As shown in Fig 6D, no significant differences were seen between naive control groups and infected test groups in both strains of mice; therefore, ARG activities were not changed during *L*. *tarentolae* infection in both strains of mice. Similar to *L*. *tropica* infections, in C57BL/6 mice the ARG activities in both naive control and test groups were considerably higher than in BALB/c mice.

### Correlation analysis between parasite burden and ARG activity

**Fig 7:**
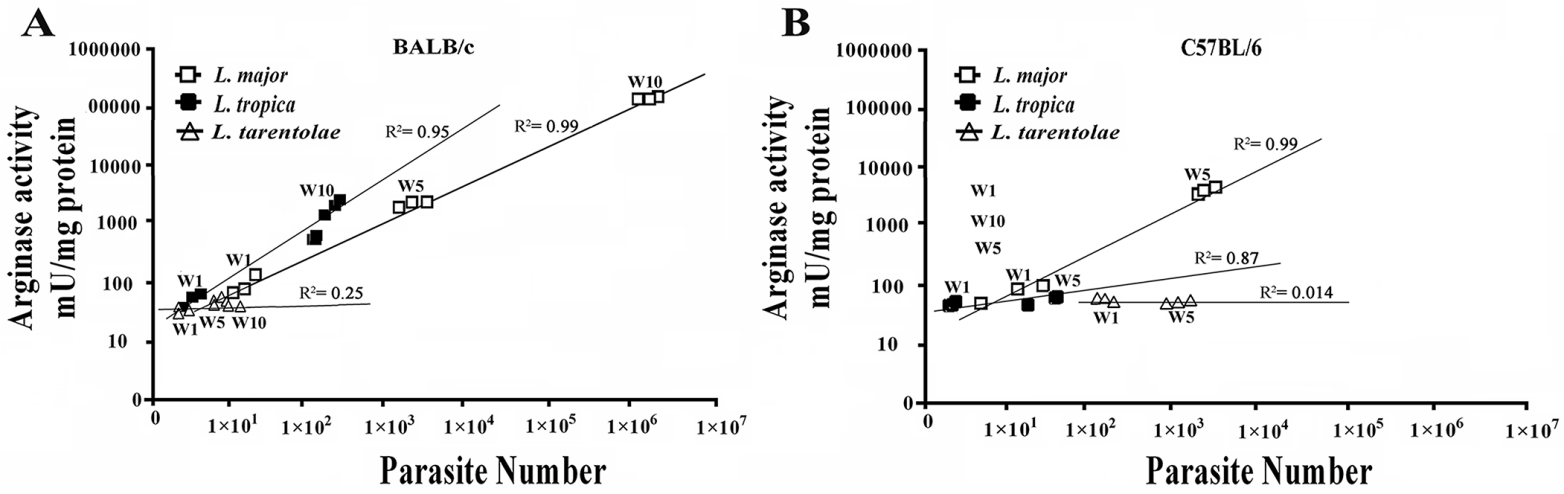
Correlation analysis between parasite numbers and ARG activity in infected draining lymph nodes (LN). At various times after infection (A) BALB/c and (B) C57BL/6 mice infected with pathogenic (*L*. *major*: 2×10^6^ parasites/ml and *L*. *tropica*: 2×10^7^ parasites/ml) and non-pathogenic *Leishmania* (*L*. *tarentolae*: 2×10^7^ parasites/ml*)* promastigotes were sacrificed, and parasite numbers as well as ARG activity in the draining LNs was determined. Data reported are those of duplicate samples, and the experimental procedure was repeated at least two times with similar outcomes. Error bars are SD (**p≤ 0.01, ****p≤ 0.001 and *****p≤ 0.0001) (W: Week).

Correlation analysis between parasite burden and ARG activity in *L*. *major* infected LNs elucidated a strong positive correlation between parasite number and ARG activities in infected BALB/c mice 10 weeks post infection (p ≤ 0.0001, r = 0.9987 and R^2^ = 0.9974) (Fig 7A), whereas a direct correlation was seen in C57BL/6 mice until 5 weeks post infection (Fig 7B). There was no correlation at 10 weeks post infection in resistant mice because the parasite number and lesion size were significantly decreased (Fig 7B). Similarly, *L*. *tropica* infected LNs showed a strong positive correlation between parasite number and ARG activities in infected BALB/c mice 5 and 10 weeks post infection (p ≤ 0.0001, r = 0.9784and R^2^ = 0.9573) (Fig 7A). Data showed that there was a direct correlation between the parasite number and the ARG activity in the lesions of C57BL/6 mice up to 5 weeks post infection (p ≤ 0.01, r = 0.9326 and R^2^ = 0.8697) (Fig 7B). Our results and others clearly have shown that parasite numbers directly correlate with ARG activities and modulate the outcome of pathogenic *L*. *major* and *L*. *tropica* infections in mice [12, 56]. As shown in Fig 7, no correlation between the parasite number and the ARG activity was seen in *L*. *tarentolae* infected LNs in both BALB/c and C57BL/6 mice.

## Discussion

The most remarkable structural signature of the Old and New World *Leishmania* ARG are the C-terminal tri-peptides AKL and SKL, respectively. They have now been genetically confirmed as the peroxisomal targeting signal (PTS-1) that serves as the topogenic signal for targeting ARG to the unique *Leishmania* glycosome [44]. *Leishmania* glycosome, a peroxisome like organelle, was found only in kinetoplastids, and the presence of this organelle shows one of the major differences among the *Leishmania* parasites and the host. da Silva et al have shown that the appropriate localization of *Leishmania* ARG in this unique organelle (glycosome) is a key factor for ARG activity and proper infectivity. In addition, they showed that dislocated ARG in *Leishmania* interrupted its infectivity. Therefore, glycosome is vital for enzyme activity in *Leishmania* parasites [21]. Our sequence analysis of *Leishmania* PTS-1 showed no equivalent PTS-1 sequence among 31 ARG family members [18, 44, 57–59]. In our investigation, all sequenced *Leishmania* spp. have this unique glycosomal signature and share a high level of homology by reference genes amino acid residues in those areas critical to enzymatic function. The non-pathogenic *L*. *tarentolae* ARG homologue shows lower sequence homology to the closest orthologues in *L*. *major*. There are some differences that may be due to the fact that *L*. *tarentolae* is a *Leishmania*-like parasite of lizards that belong to the genus *Sauroleishmania* and partly distinct from *Leishmania* genus.

Our data demonstrated that all pathogenic and non-pathogenic *Leishmania* express their own *arg* gene, and have ARG activity. According to previous studies on ARG deleted mutants, it is a vital gene in *Leishmania* parasites [12, 18, 20, 56, 60]. One interesting new finding is that, in all three tested *Leishmania* species, the ARG activity at both enzymatic and transcriptomic levels in the logarithmic growth phase of the parasites is higher than that in the stationary one. Although the exact mechanisms of higher ARG activity in the logarithmic growth phase is unknown, the possible explanation can be the higher replication rate of the parasites and their requirement for particular essential needs at this stage. Also, ARG, is an essential enzyme in *Leishmania* promastigotes [18, 47], the higher expression in the logarithmic stage growth phase coincides with the major roles of this enzyme in L-arginine metabolism and polyamines biosynthesis. At stationary growth phase, *Leishmania* is ready to infect the host cells and may need less metabolite in compare to logarithmic phase. Thus, it is interesting to characterize all key biochemical and immunological pathways such as ARG activity in the pathogenic and non-pathogenic *Leishmania* species at different stages.

This study showed that in *Leishmania* infected MQs, the activity of ARG were increased over uninfected; therefore, parasite infection indirectly upregulated the ARG activity at *in vitro* condition. *Leishmania* parasites may partly activate ARGs, inactivate NO production by host cells, and enhance parasite survival via local depletion of the iNOS substrate L-arginine and reduced NO levels [23]. High ARG activity will limit substrate availability for iNOS and macrophages cannot control efficient killing of intracellular *Leishmania* and they may also serve as long-term host cells that facilitate the replication of the parasites [23]. One previous study revealed that *Leishmania* proliferation inside macrophages was remarkably diminished in the absence of *Leishmania* ARG and therefore, ARG deficient parasites were unable to induce host ARG that are in agreement with our study [12]. Interestingly, when the human THP1 cells were infected with different *Leishmania* species, no significant differences in the activity of ARG were found between the pathogenic species and non-pathogenic *L*. *tarentolae*. This suggests that ARG is essential for all species of *Leishmania* and they need ARG to survive and multiply inside host cells [17, 48].

A direct correlation was seen between ARG activities and NO production. Since *Leishmania* parasites are inside the phagolysosome in infected macrophages, their ARG activity is compartmentalized in their glycosome. Suboptimally activated macrophages due to low concentrations of IFN-γ, iNOS and ARG stay partly inactivated, allowing *Leishmania* parasites to take up and utilize accessible arginine for their multiplication and proliferation [12, 23, 61]. Actually, there is challenging competition for the L-arginine between ARG and iNOS; it is possible that parasite ARG utilizes the host arginine resource and as a result reduce its availability for iNOS activity therefore, resulting in decreased NO production.

We report here that infection of susceptible BALB/c mice with pathogenic *L*. *major* leads to non-healing infection and increased parasite load in the LN as well as significantly enhanced ARG activity at the site of infection and in the draining LN. In contrast, infection of genetically resistant C57BL/6 mice with pathogenic *L*. *major* resulted in healed lesions with low ARG activity and reduced parasite load in the LN. Studies have shown that the ARG activity of both host and the parasites is important for the replication of *L*. *major* in *in vivo* [56, 62]. In sharp contrast, C57BL/6 mice controlled both the lesion and the *Leishmania* replication; therefore, it resulted in decreased ARG activity [56]. Although the pathology, control and replication of the *Leishmania* parasites in resistant C57BL/6 and susceptible BALB/c mice infected with *L*. *major* have been attributed to polarized Th1/Th2 responses [56, 63], the exact mechanism of action is not completely understood. Plausible explanations for this propensity are effects of genetic background differences in both strains of mice. Studies have shown that uninfected or intact macrophages from BALB/c mice are more apt to upregulation of ARG than those from C57BL/6 mice [56]. Macrophages of susceptible mice give a better “milieu” for replication of the *Leishmania* than do resistance mice macrophages. Therefore, in these milieu susceptible mice permits the parasites to express their antigens effectively to reach at the threshold of inhibiting effective cellular immune responses [56].

Published data using mouse models of leishmaniasis caused by *L*. *tropica* are scarce; therefore, this is the first report showing the ARG activity in pathogenic *L*. *tropica* promastigotes that were used to initiate infections in BALB/c and C57BL/6 mice. In both strains of mice, the lesions grew non-progressively and non-ulceratively, parasite replication was slow to develop and remained low. Although the level of ARG activity in both mice were lower than in *L*. *major* infected mice, ARG activity in BALB/c mice were higher than C57BL/6 mice. These results are in agreement with those obtained by Anderson et al., where they showed that dermal lesions in the ears of BALB/c and C57BL/6 mice were slow to develop and displayed minimal skin pathology [53]. A potential explanation for this may be due to the fact that the inflammatory responses in cutaneous lesions of *L*. *tropica* infections develop slower as compared to that of *L*. *major* [53].

*L*. *tarentolae* is a non-pathogenic parasite that does not cause pathology in humans nor in immunodeficient mice and its non-pathogenicity has been recently proven in several studies [55, 64, 65]. We showed here that injection of *L*. *tarentolae* did not induce any swelling in either BALB/c or C57BL/6 mice. Although *L*. *tarentolae* can enter into macrophages, they could not induce ARG activity at the infection site and the draining LNs. Interestingly; we found that in LNs of C57BL/6 mice (about 400 parasites/LN) more surviving *L*. *tarentolae* than in LNs of BALB/c mice (about 20 parasites/LN). However, there was no significant difference in ARG activities in infected mice, a low number of *L*. *tarentolae* parasites replicated in the draining LN of C57BL/6 mice and the parasite numbers reached to background levels at the end of the experiment. Although *L*. *tarentolae* can enter macrophages and differentiate into intracellular amastigote forms, there are no clear evidences for their effective replication inside the macrophages [55, 65]. The exact mechanism of why parasite can replicate in macrophages of LNs of C57BL/6 mice but not replicate in the macrophages of BALB/c mice is not completely clear and needs further investigation.

Further research is required to assess whether ARG activity is a pivotal element in the outcome of leishmaniasis in humans, especially in the acute and chronic lesions [66], and it is very important to compare the ARG activity between lesions derived and attenuated *Leishmania* species/strains. Another interesting experiment would be to overexpress ARG in different *Leishmania* species/strains and test the effects on infectivity of the disease. More studies are required to clarify the contribution of host and parasite ARG activity to the course of cutaneous leishmaniasis [56]. One of the most important limitations of our work is the quantification of ARG activity in purified amastigotes isolated from infected human macrophages; therefore, it is suggested to measure the ARG activity in the purified amastigotes of parasites and in THP1 cells separately.

In summary, *Leishmania*-derived ARG is a potentially vital enzyme in promastigotes that regulates parasite growth. Furthermore, *Leishmania* promote their own existence via manipulating the host’s intracellular signaling pathways to repress different enzymatic processes. Therefore, we consider that our work may open new perspectives for the study of ARG in both mammalian host as a favorable environment for parasite growth and in *Leishmania* parasites as a potential target for novel therapeutic and vaccine research. Thus, determining the main role of ARG in *Leishmania* provides valuable knowledge that directs to a better understanding of host/parasite interactions and to identification of the efficient targets for vaccination and importantly for their treatments.

## Supporting information

S1 Fig A multiple alignment of ARG proteins between *Leishmania* parasites and mammalian. ARG protein sequences from human, mice and various *Leishmania* species were aligned. The C-terminal tripeptide (SKL and AKL) signature for glycosomal localization in *Leishmania* parasites is highlighted with the rectangle box. The alignment was applied by using Clustal W and BioEdit Sequence Alignment Editor Softwares. Stars indicate fully conserved residues; colons indicate highly conserved residues; and periods indicate weakly conserved residues. The sequences analysed are: The retrieved GenBank nucleotide sequences analysed are: *Homo sapiens* ARG 1 (NP_001231367.1), *Homo sapiens* ARG 2 (NP_001163.1), *Mus musculus* ARG 1 (U51805.1), *Mus musculus* ARG 2 (U90886.1), *L*. *panamensis* MHOM/PA/94/PSC-1 (XM_010704259), *L*. *mexicana* MNYC/BZ/62/M379 (AY386701.1), *L*. *major* Friedlin LMJF_35_1480 (XM_003722493.1), *L*. *infantum* JPCM5 (XM_001468931.1), *L*. *donovani* LDBPK_351490 (XM_003864686.1), *L*. *braziliensis* MHOM/BR/75/M2904 (XM_001568200.1) and *L*. *amazonensis* MHOM/BR/1973/M2269 (AF038409.2). Sequenced *Leishmania* arg genes in the current study are shown in black doted as following: *L*. *tropica* MOHM/IR/09/Khamesipour-Mashhad (KU641753), *L*. *major* MRHO/IR/75/ER (KU641750) and *L*. *tarentolae* Tar II ATCC30267 (KU641752).
(TIF)

S2 Fig Phylogenetical relationships between old and new world *Leishmania* spp. and mammalian *arg* genes using maximum likelihood (ML) algorithm. The written numbers next to the each branch are computed from bootstrap values of 500 replicates. The evolutionary tree was created by user-friendly MEGA 5.05. The retrieved GenBank nucleotide sequences analysed are: *Homo sapiens* ARG 1 (NP_001231367.1), *Homo sapiens* ARG 2 (NP_001163.1), *Mus musculus* ARG 1 (U51805.1), *Mus musculus* ARG 2 (U90886.1), *L*. *panamensis* MHOM/PA/94/PSC-1 (XM_010704259), *L*. *mexicana* MNYC/BZ/62/M379 (AY386701.1), *L*. *major* Friedlin LMJF_35_1480 (XM_003722493.1), *L*. *infantum* JPCM5 (XM_001468931.1), *L*. *donovani* LDBPK_351490 (XM_003864686.1), *L*. *braziliensis* MHOM/BR/75/M2904 (XM_001568200.1) and *L*. *amazonensis* MHOM/BR/1973/M2269 (AF038409.2). Sequenced *Leishmania* arg genes in the current study are shown in black doted as following: *L*. *tropica* MOHM/IR/09/Khamesipour-Mashhad (KU641753), *L*. *major* MRHO/IR/75/ER (KU641750) and *L*. *tarentolae* Tar II ATCC30267 (KU641752).
(TIF)
