# Supplementary figures and images for: Arginase activity in pathogenic and non-pathogenic species of Leishmania parasites
Source: PLoS Negl Trop Dis. 2017 Jul 14;11(7):e0005774. doi: 10.1371/journal.pntd.0005774 (PMC5529023; doi:10.1371/journal.pntd.0005774)

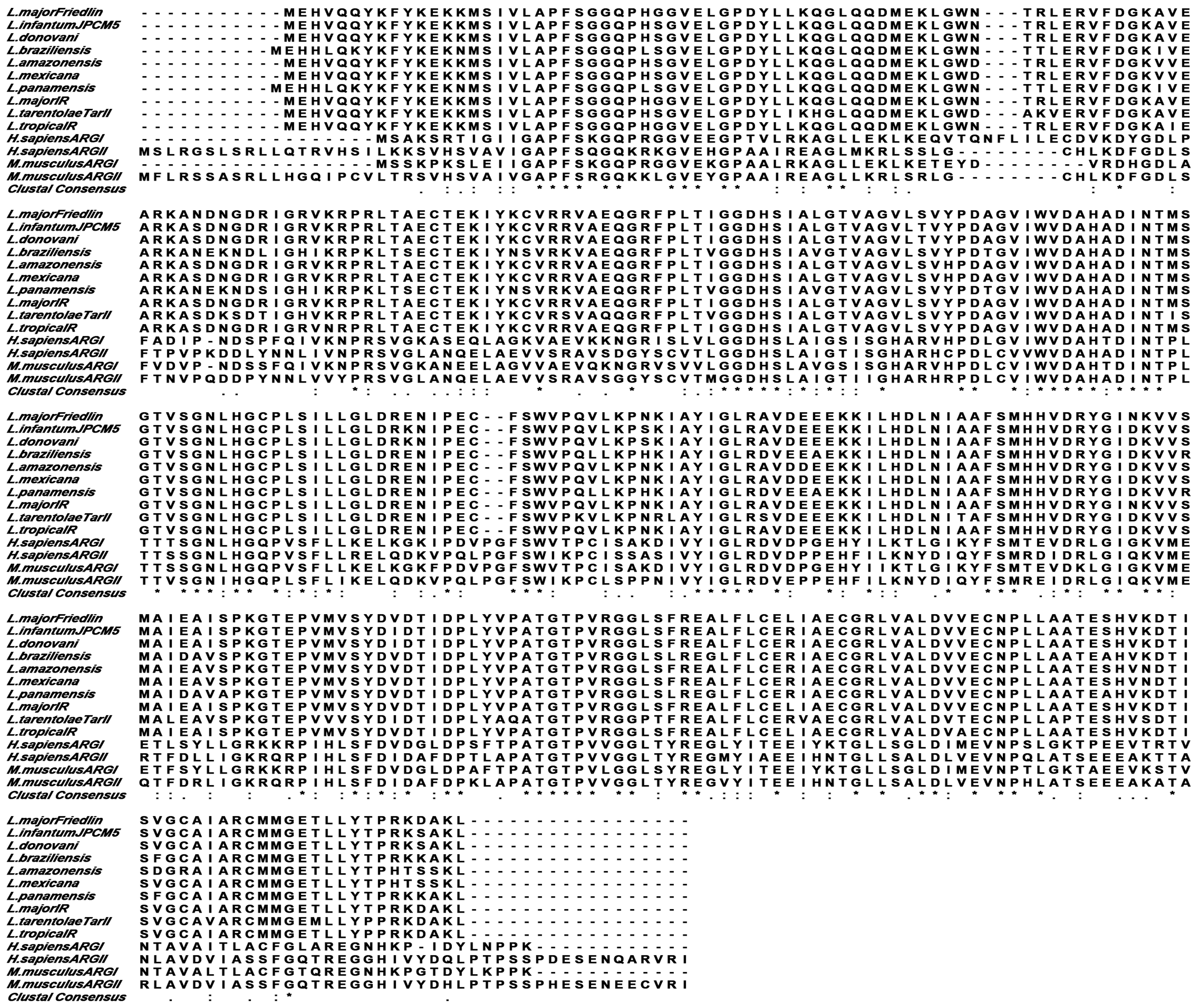

Supplement: S1 Fig — ARG protein sequences from human, mice and various Leishmania species were aligned. The C-terminal tripeptide (SKL and AKL) signature for glycosomal localization in Leishmania parasites is highlighted with the rectangle box. The alignment was applied by using Clustal W and BioEdit Sequence Alignment Editor Softwares. Stars indicate fully conserved residues; colons indicate highly conserved residues; and periods indicate weakly conserved residues. The sequences analysed are: The retrieved GenBank nucleotide sequences analysed are: Homo sapiens ARG 1 (NP_001231367.1), Homo sapiens ARG 2 (NP_001163.1), Mus musculus ARG 1 (U51805.1), Mus musculus ARG 2 (U90886.1), L. panamensis MHOM/PA/94/PSC-1 (XM_010704259), L. mexicana MNYC/BZ/62/M379 (AY386701.1), L. major Friedlin LMJF_35_1480 (XM_003722493.1), L. infantum JPCM5 (XM_001468931.1), L. donovani LDBPK_351490 (XM_003864686.1), L. braziliensis MHOM/BR/75/M2904 (XM_001568200.1) and L. amazonensis MHOM/BR/1973/M2269 (AF038409.2). Sequenced Leishmania arg genes in the current study are shown in black doted as following: L. tropica MOHM/IR/09/Khamesipour-Mashhad (KU641753), L. major MRHO/IR/75/ER (KU641750) and L. tarentolae Tar II ATCC30267 (KU641752). (TIF) [file pntd.0005774.s001.tif]

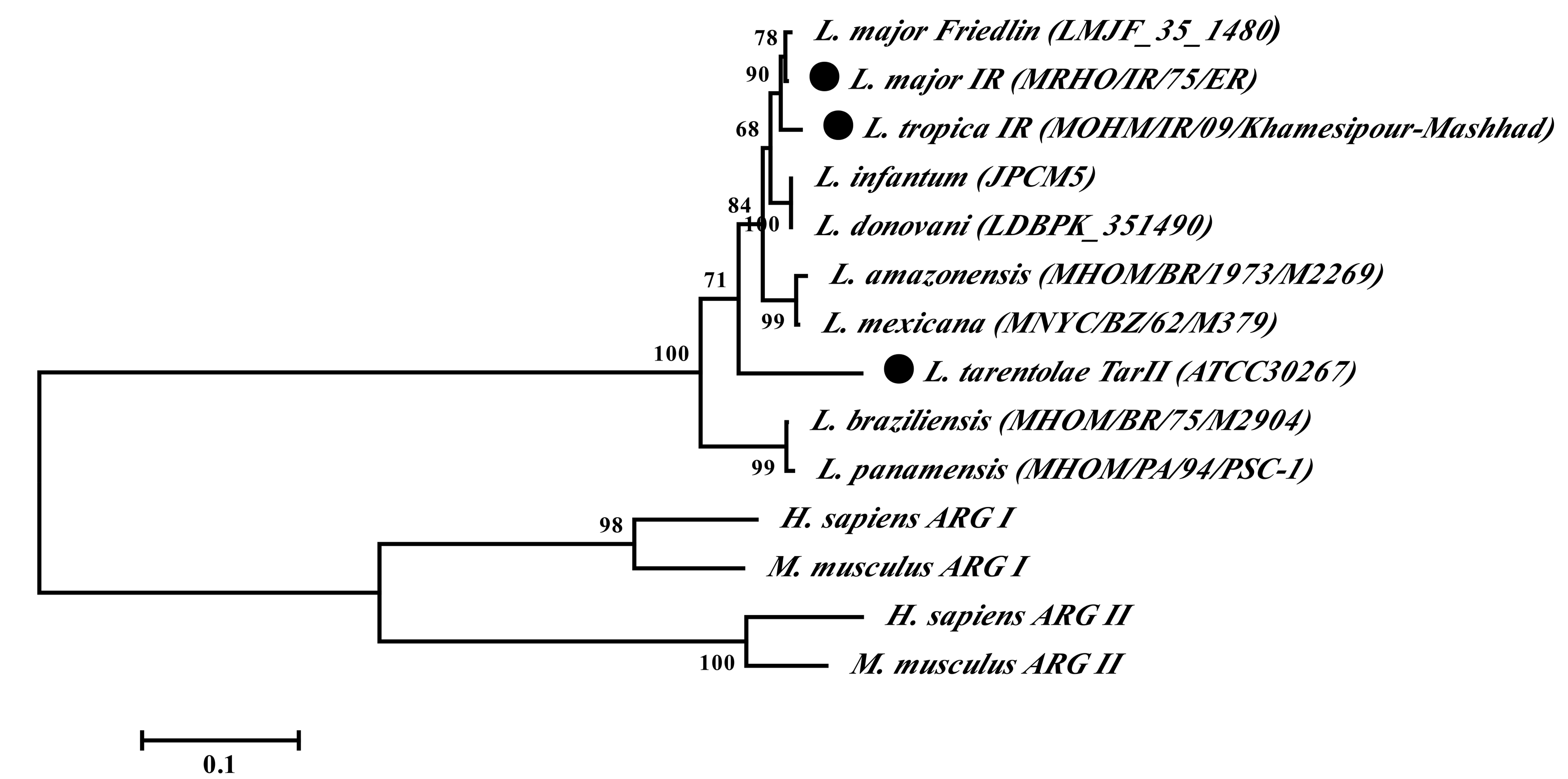

Supplement: S2 Fig — The written numbers next to the each branch are computed from bootstrap values of 500 replicates. The evolutionary tree was created by user-friendly MEGA 5.05. The retrieved GenBank nucleotide sequences analysed are: Homo sapiens ARG 1 (NP_001231367.1), Homo sapiens ARG 2 (NP_001163.1), Mus musculus ARG 1 (U51805.1), Mus musculus ARG 2 (U90886.1), L. panamensis MHOM/PA/94/PSC-1 (XM_010704259), L. mexicana MNYC/BZ/62/M379 (AY386701.1), L. major Friedlin LMJF_35_1480 (XM_003722493.1), L. infantum JPCM5 (XM_001468931.1), L. donovani LDBPK_351490 (XM_003864686.1), L. braziliensis MHOM/BR/75/M2904 (XM_001568200.1) and L. amazonensis MHOM/BR/1973/M2269 (AF038409.2). Sequenced Leishmania arg genes in the current study are shown in black doted as following: L. tropica MOHM/IR/09/Khamesipour-Mashhad (KU641753), L. major MRHO/IR/75/ER (KU641750) and L. tarentolae Tar II ATCC30267 (KU641752). (TIF) [file pntd.0005774.s002.tif]
